# Cycle threshold values are inversely associated with poorer outcomes in hospitalized patients with COVID-19: a prospective, observational cohort study conducted at a UK tertiary hospital

**DOI:** 10.1016/j.ijid.2021.08.022

**Published:** 2021-10

**Authors:** Jenny Wright, Felix Achana, Lavanya Diwakar, Malcolm G. Semple, Will D. Carroll, Kenneth Baillie, Christopher Thompson, Alice Alcock, Timothy S. Kemp

**Affiliations:** a University Hospitals of North Midlands NHS Trust, Stoke-on-Trent, UK; b University of Oxford, Oxford, UK; c University of Liverpool, Liverpool, UK; d University of Edinburgh Roslin Institute, Edinburgh, UK

**Keywords:** COVID-19, Respiratory infection, Viral infection

## Abstract

This single-centre observational study demonstrated that lower cycle threshold (Ct) values (indicating higher viral loads) on admission to hospital were associated with poorer outcomes in unvaccinated, hospitalized patients with coronavirus disease 2019 (COVID-19). Demographic and outcome data were collected prospectively for all adult patients who tested positive for severe acute respiratory syndrome coronavirus-2 on admission to the University Hospitals North Midlands NHS Trust between 1 February and 1 July 2020. Nasopharyngeal swab samples were obtained, and a valid Ct value was determined for all patients using the Viasure reverse transcription polymerase chain reaction assay, validated by Public Health England, on admission to hospital. Multi-variable logistic regression results based on data from 618 individuals demonstrated a significant inverse relationship between the odds of death and Ct values (adjusted odds ratio 0.95, 95% confidence interval 0.92–0.98, *P*=0.001). The association remained highly significant after adjusting for known clinical risk factors for COVID-19

## Highlights

• A multi-variable regression analysis of data for hospitalized patients with coronavirus disease 2019 was undertaken.
• The likelihood of death and cycle threshold (Ct) values at admission were studied.
• Adjustments were made for known clinical risk factors for the disease.
• Lower Ct values were associated with poorer outcomes in hospitalized patients.

## Introduction

Clinicians need to identify patients with coronavirus disease 2019 (COVID-19) with a higher risk of poor outcome and mortality at an early stage of hospital admission. This prospective, observational cohort study was conducted at a UK tertiary care hospital to determine the relationship between the likelihood of death and cycle threshold (Ct) values in an unvaccinated UK population with COVID-19 on admission to hospital. Statistical adjustment was made for other known risk factors associated with poor outcome. Ct values are semi-quantitative values which are inversely proportional to the viral load in a reverse transcription polymerase chain reaction (PCR) test for severe acute respiratory syndrome coronavirus-2 (SARS-CoV-2).

## Methods

Demographic and outcome data were collected prospectively for all adult patients who tested positive for SARS-CoV-2 on admission to the University Hospitals North Midlands (UHNM) NHS Trust between 1 February and 1 July 2020 using the ISARIC World Health Organization (WHO) Clinical Characterisation Protocol (Docherty et al., 2020). Nasopharyngeal swab samples were obtained, and valid Ct values were determined for all patients using the Viasure reverse transcription PCR assay, validated by Public Health England, on admission to hospital. Ct values <38 were considered positive. Viasure targets the *ORF1ab* (Target 1) and *N* genes (Target 2) of the SARS-CoV-2 viral genome (Public Health England, 2020). Target 1 was chosen as the primary exposure of interest as this is specific to the SARS-CoV-2 viral genome. Ct values were combined with ISARIC data for statistical analysis.

Multi-variable logistic regression was used to determine the association between Ct value and death within 28 days of a positive SARS-CoV-2 PCR test. Adjustments were made for age, gender, ethnicity, obesity, cardiovascular disease, chronic pulmonary disease, chronic kidney disease and diabetes. These covariates were selected *a priori* based on current understanding of the factors predicting poor outcomes in hospital cases of COVID-19 (Knight et al., 2020). Statistical significance was assessed at the 5% significance level and results are presented as odds ratios and 95% confidence intervals (CI). Analyses were conducted in R Version 4.0.1.

## Results

**Figure 1:**
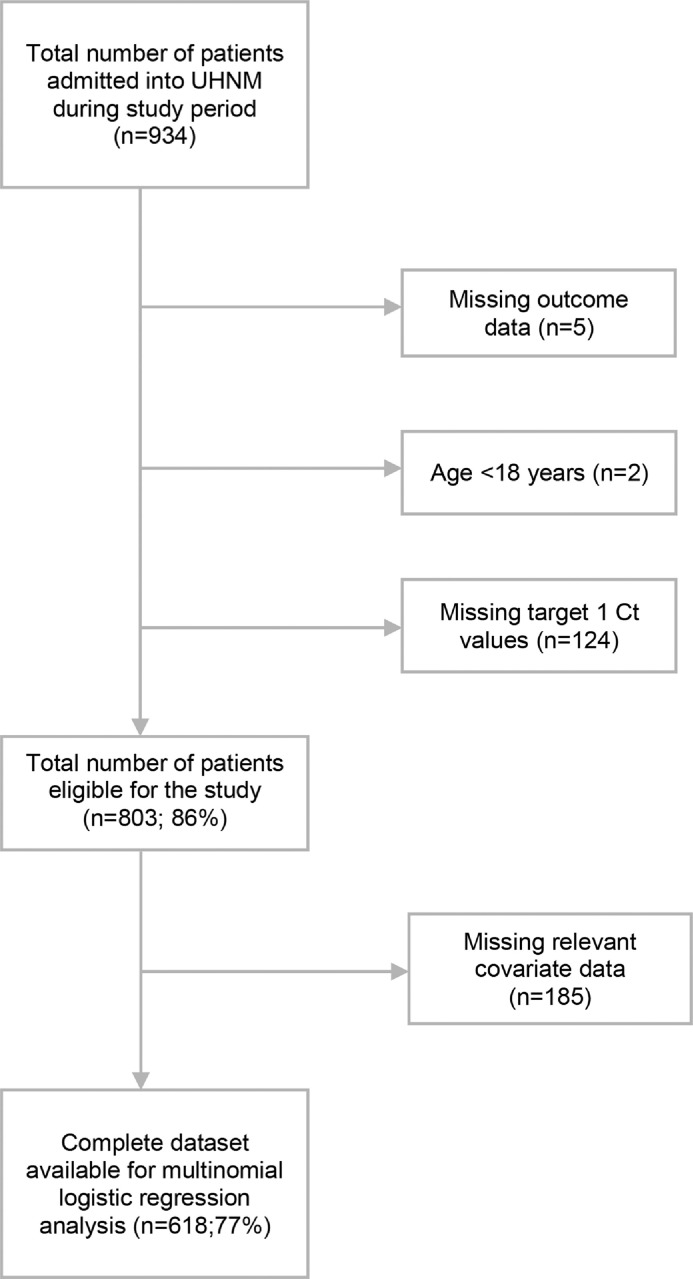
Flow diagram illustrating how the final study sample size was determined. UHNM, University Hospitals North Midlands NHS Trust; Ct, cycle threshold.

In total, 803 SARS-CoV-2-positive adults (>18 years of age) with a valid Ct value determined on admission to hospital were eligible for inclusion in this study, and were followed-up for a period of up to 28 days (Figure 1). The median age was 77 years (range 19–100 years), 55% were male and 91% were of white ethnicity. Thirty-five percent of participants had a history of cardiovascular disease, 18% had chronic pulmonary disease, 19% had chronic kidney disease, 14% were asthmatic, 13% had either complicated or uncomplicated diabetes, and 7% were obese. The median Ct value for Target 1 was 25.8 (interquartile range 21.3–30.0). In total, 285 of 803 patients (35.5%) died within 28 days of a positive SARS-CoV-2 PCR result.

Multi-variable logistic regression results based on data from 618 (77.0%) individuals with complete covariate information are presented in Table 1. There was a significant inverse relationship between the probability of death and Ct value [adjusted odds ratio (aOR) 0.95, 95% CI 0.92–0.98; *P*=0.001]. The only other variables that were significantly associated with mortality were age (aOR 1.05, 95% CI 1.03–1.06; *P*<0.001) and diabetes (aOR 1.73, 95% CI 1.01–2.97; *P*=0.044).

**Table 1:** Multi-variable logistic regression results based on complete case analysis.

	Death		Death or continuous hospitalization	
Variable	Odds ratio (95% CI)	*P*-value	Odds ratio (95% CI)	*P*-value
Age (years)	1.05 (1.03–1.06)	<0.001	1.04 (1.02–1.05)	<0.001
Male	1.43 (1.00–2.07)	0.052	1.2 (0.85–1.69)	0.308
Non-white	0.66 (0.15–2.21)	0.539	0.77 (0.23–2.27)	0.65
Cardiovascular disease	1.00 (0.68–1.47)	0.992	0.95 (0.65–1.39)	0.799
Chronic pulmonary disease	1.17 (0.75–1.82)	0.475	1.17 (0.76–1.82)	0.469
Chronic kidney disease	1.22 (0.78–1.91)	0.382	1.51 (0.97–2.36)	0.071
Diabetes	1.68 (1.03–2.75)	0.037	1.35 (0.83–2.22)	0.227
Obesity	1.33 (0.68–2.53)	0.39	1.1 (0.59–2.05)	0.764
Cycle threshold	0.95 (0.92–0.98)	0.001	0.94 (0.91–0.97)	<0.001
Constant	0.01 (0.00–0.04)	<0.001	0.05 (0.02–0.12)	<0.001

CI, confidence interval.

Sensitivity analyses carried out after imputing missing data produced results consistent with the complete case analysis for both outcomes. Similar results were obtained when Target 2 was used in the analysis instead of Target 1 (data available on request).

In this prospective, observational study carried out in a tertiary UK hospital, lower Ct values (indicating higher viral loads) were associated with poorer outcomes in hospitalized patients with COVID-19. This association remained highly significant after adjusting for known clinical risk factors for the disease. High viral loads are associated with adverse outcomes in human immunodeficiency virus and Ebola infections (Fitzpatrick et al., 2015; Li et al., 2015). There have been reports that higher viral loads are associated with adverse outcomes in patients with COVID-19 in China, Taiwan and Brazil (Choudhuri et al., 2020; Faíco-Filho et al., 2020; Huang et al., 2020; Liu et al., 2020; Yu et al., 2020), but none from the UK.

## Discussion

The main strength of this study is that it used a validated, generalizable dataset for analysis. Ct values were measured by validated methods. Robust statistical methods were used to account for interactions and missing data. However, the study has several limitations. It was limited by its relatively small size. Therefore, the analysis was restricted to eight covariates known to be associated with poorer outcomes. Whilst this reduced the risk of confounding due to multiple hypothesis testing, it did not allow exploration of the data to determine whether other measurements, such as admission cardiovascular status, oxygen saturation and liver failure or need for mechanical ventilation were associated with death. However, a logistic regression model incorporating all of these measurements did not show any significant associations. Moreover, the data are limited to a single tertiary care centre in the UK, and the population characteristics may vary in relation to other areas. It is also unclear if these associations will remain significant after accounting for therapeutic agents (e.g. dexamethasone, anti-IL-6 treatment) in COVID-19 management. These findings suggest that Ct values may have the potential to help clinicians identify patients at high risk of mortality from COVID-19 at the point of hospital admission. It is suggested that the analysis should be repeated using a larger, multi-centre dataset across different time periods.
